# Supplementary material for: Algorithmic bias in social research: A meta-analysis
Source: PLoS One. 2020 Jun 8;15(6):e0233625. doi: 10.1371/journal.pone.0233625 (PMC7279593; doi:10.1371/journal.pone.0233625)
Supplement: S1 Appendix — (PDF) [file pone.0233625.s001.pdf]

Appendix A1: List of reanalyzed QCA articles

#	Journal Article	Label
1	Ackrén, Maria, and Pär M. Olausson. 2008. "Condition(s) for island autonomy." *International Journal on Minority and Group Rights* 15 (2-3):227-58.	ackren_olausson_2008
2	Adhikari, Prakash, and Steven Samford. 2013. "The Nepali state and the dynamics of the Maoist insurgency." *Studies in Comparative International Development* 48 (4):457-81.	adhikari_samford_2013
3	Albala, Adrián. 2017. "Bicameralism and coalition cabinets in presidential polities: A configurational analysis of the coalition formation and duration processes." *British Journal of Politics and International Relations* 19 (4):735-54.	albala_2017
4	Alijla, A., and M. Aghdam. 2017. "Different paths to democracy in the MENA region: A configurational comparative analysis." *Journal of Political Sciences & Public Affairs* 5 (2):265.	alijla_aghdam_2017
5	Allen, Matthew M. C., and Maria L. Aldred. 2011. "Varieties of capitalism, governance, and high-tech export performance: A fuzzy-set analysis of the new EU member states." *Employee Relations* 33(4):334-55.	allen_aldred_2011
6	Amenta, Edwin, and Thomas A. Elliott. 2017. "All the right movements? Mediation, rightist movements, and why US movements received extensive newspaper coverage." *Social Forces* 96 (2):803-30.	amenta_elliott_2017
7	Ansorg, Nadine. 2014. "Wars without borders: Conditions for the development of regional conflict systems in sub-Saharan Africa." *International Area Studies Review* 17 (3):295-312.	ansorg_2008
8	Avdagic, Sabina. 2010. "When are concerted reforms feasible? Explaining the emergence of social pacts in Western Europe." *Comparative Political Studies* 43 (5):628-57.	avdagic_2010
9	Bakker, René, Bart Cambré, Leonique Korlaar, and Joerg Raab. 2011. "Managing the project learning paradox: A set-theoretic approach toward project knowledge transfer." *International Journal of Project Management* 29 (5):494-503.	bakker_et_al_2011
10	Bank, André, Thomas Richter, and Anna Sunik. 2015. "Long-term monarchical survival in the Middle East: A configurational comparison, 1945-2012." *Democratization* 22 (1):179-200.	bank_et_al_2015
11	Bara, Corinne. 2014. "Incentives and opportunities: A complexity-oriented explanation of violent ethnic conflict." *Journal of Peace Research* 51 (6):696-710.	bara_2014
12	Basedau, Matthias, and Thomas Richter. 2014. "Why do some oil exporters experience civil war but others do not? Investigating the conditional effects of oil." *European Political Science Review* 6 (4):549-74.	basedau_richter_2014

13 Befani, Barbara. 2005. "The mechanisms of suicide: A realist approach." *International Review of Sociology* 15 (1):51-75. befani_2005

14 Berg-Schlosser, Dirk. 2008. "Determinants of democratic successes and failures in Africa." *European Journal of Political Research* 47 (3):269-306. bergschlosser_2008

15 Berg-Schlosser, Dirk, and Gisèle De Meur. 1994. "Conditions of democracy in interwar Europe: A Boolean test of major hypotheses." *Comparative Politics* 26 (3):253-79. bergschlosser_demeur_1994

16 Berlin, Mark S. 2016. "Why (not) arrest? Third-party state compliance and noncompliance with international criminal tribunals." *Journal of Human Rights* 15 (4):509-32. berlin_2016

17 Binder, Martin. 2015. "Paths to intervention: What explains the UN's selective response to humanitarian crises?" *Journal of Peace Research* 52 (6):712-26. binder_2015

18 Blake, Charles H. 1996. "The politics of inflation-fighting in new democracies." *Studies in Comparative International Development* 31 (2):37-57. blake_1996

19 Bochsler, Daniel. 2011. "It is not how many votes you get, but also where you get them. Territorial determinants and institutional hurdles for the success of ethnic minority parties in post-communist countries." *Acta Politica* 46 (3):217-38. bochsler_2011

20 Bochsler, Daniel. 2012. "When two of the same are needed: A multilevel model of intragroup ethnic party competition." *Nationalism and Ethnic Politics* 18 (2):216-41. bochsler_2012

21 Böhm, Eva, Andreas Eggert, and Christoph Thiesbrummel. 2017. "Service transition: A viable option for manufacturing companies with deteriorating financial performance?" *Industrial Marketing Management* 60:101-11. bohm_et_al_2017

22 Borgna, Camilla. 2016. "Multiple paths to inequality. How institutional contexts shape the educational opportunities of second-generation immigrants in Europe." *European Societies* 18 (2):180-99. borgna_2016

23 Botta, Marco, and Guido Schwellnus. 2015. "Enforcing state aid rules in EU candidate countries: A qualitative comparative analysis of the direct and indirect effects of conditionality." *Journal of European Public Policy* 22 (3):335-52. botta_schwellnus_2015

24 Bretthauer, Judith M. 2015. "Conditions for peace and conflict: Applying a fuzzy-set qualitative comparative analysis to cases of resource scarcity." *Journal of Conflict Resolution* 59 (4):593-616. bretthauer_2015

25 Breuer, Anita. 2009. "The use of government-initiated referendums in Latin America: Towards a theory of referendum causes." *Revista De Ciencia Politica* 29 (1):23-55. breuer_2009

26 Brueggemann, John, and Terry Boswell. 1998. "Realizing solidarity: Sources of interracial unionism during the Great Depression." *Work and Occupations* 25 (4):436-82. brueggemann_boswell_1998

27 Cacciatore, Federica, Alessandro Natalini, and Claudius Wagemann. 2015. "Clustered Europeanization and national reform programmes: A qualitative comparative analysis." *Journal of European Public Policy* 22 (8):1186-211. cacciatore_et_al_2015

28 Cárdenas, Julián. 2012. "Varieties of corporate networks: Network analysis and fsQCA." *International Journal of Comparative Sociology* 53 (4):298-322. cardenas_2012

29 Cassani, Andrea, Francesca Luppi, and Gabriele Natalizia. 2016. "Pathways of democratisation to human development in post-communist countries." *European Journal of Political Research* 55 (3):512-30. cassani_et_al_2016

30 Castillo Ortiz, Pablo José, and Iván Medina. 2016. "Paths to the recognition of homoparental adoptive rights in the EU-27: A QCA analysis." *Contemporary Politics* 22 (1):40-56. castilloortiz_medina_2016

31 Cebotari, Victor, and Maarten P. Vink. 2013. "A configurational analysis of ethnic protest in Europe." *International Journal of Comparative Sociology* 54 (4):298-324. cebotari_vink_2013

32 Chaebo, Gemael, and Janann J. Medeiros. 2017. "Conditions for policy implementation via co-production: The control of dengue fever in Brazil." *Public Management Review* 19 (10):1381-98. chaebo_medeiros_2017

33 Chan, Steve. 2003. "Explaining war termination: A Boolean analysis of causes." *Journal of Peace Research* 40 (1):49-66. chan_2003

34 Chandra Balodi, Krishna. 2016. "Configurations and entrepreneurial orientation of young firms: Revisiting theoretical specification using crisp-set qualitative comparative analysis." *Management Decision* 54 (4):1004-19. chandrabalodi_2016

35 Christmann, Anna. 2010. "Damoklesschwert Referendum? Die indirekte Wirkung ausgebauter Volksrechte auf die Rechte religiöser Minderheiten." *Swiss Political Science Review* 16 (1):1-41. christmann_2010

36 Chung, Chi-Nien. 2001. "Markets, culture and institutions: The emergence of large business groups in Taiwan, 1950s-1970s." *Journal of Management Studies* 38 (5):719-45. chung_2001

37 Clément, Caty. 2004. "Un modèle commun d'effondrement de l'état? Une AQQC du Liban, de la Somalie et de l'ex-Yougoslavie." *Revue Internationale de Politique Comparée* 11 (1):35-50. clement_2004

38 Coverdill, James E., and William Finlay. 1995. "Understanding mills via mill-type methods: An application of qualitative comparative analysis to a study of labor management in southern textile manufacturing." *Qualitative Sociology* 18 (4):457-78. coverdill_finlay_1995

39 Cress, Daniel M., and David A. Snow. 1996. "Mobilization at the margins: resources, benefactors, and the viability of homeless social movement organizations." *American Sociological Review* 61 (6):1089-109. cress_snow_1996

40 Cress, Daniel M., and David A. Snow. 2000. "The outcomes of homeless mobilization: The influence of organization, disruption, political mediation, and framing." *American Journal of Sociology* 105 (4):1063-104. cress_snow_2000

41	Crilly, Donal. 2011. "Predicting stakeholder orientation in the multinational enterprise: A mid-range theory." *Journal of International Business Studies* 42 (5):694-717.	crilly_2011
42	Crilly, Donal, Maurizio Zollo, and Morten T. Hansen. 2012. "Faking it or muddling through? Understanding decoupling in response to stakeholder pressures." *Academy of Management Journal*, 55(6), 1429-1448.	crilly_et_al_2012
43	Cristofoli, Daniela, and Josip Markovic. 2016. "How to make public networks really work: A qualitative comparative analysis." *Public Administration* 94 (1):89-110.	cristofoli_markovic_2016
44	Csergő, Zsuzsa, Philippe Roseberry, and Stefan Wolff. 2017. "Institutional outcomes of territorial contestation: Lessons from post-communist Europe, 1989-2012." *Publius: The Journal of Federalism* 47 (4):491-521.	csergo_et_al_2017
45	Da Roit, Barbara, Marcel Hoogenboom, and Bernhard Weicht. 2015. "The gender informal care gap." *European Societies* 17 (2):199-218.	daroit_et_al_2015
46	Damonte, Alessia. 2014. "Policy tools for green growth in the EU15: A qualitative comparative analysis." *Environmental Politics* 23 (1):18-40.	damonte_2014
47	Dardanelli, Paolo. 2012. "Europeanization and the unravelling of Belgium: A comparative analysis of party strategies." *Acta Politica* 47 (2):181-209.	dardanelli_2012
48	Dardanelli, Paolo. 2014. "European integration, party strategies, and state restructuring: A comparative analysis." *European Political Science Review* 6 (2):213-36.	dardanelli_2014
49	Davidsson, Johan Bo, and Patrick Emmenegger. 2013. "Defending the organisation, not the members: Unions and the reform of job security legislation in Western Europe." *European Journal of Political Research* 52 (3):339-63.	davidsson_emmenegger_2013
50	Dawson, Gregory S., James S. Denford, and Kevin C. Desouza. 2016. "Governing innovation in U.S. State government: An ecosystem perspective." *Journal of Strategic Information Systems* 25 (4):299-318.	dawson_et_al_2016
51	Dekker, Rianne, and Peter Scholten. 2017. "Framing the immigration policy agenda." *International Journal of Press/Politics* 22 (2):202-22.	dekker_scholten_2017
52	Delreux, Tom. 2009. "The EU negotiates multilateral environmental agreements: Explaining the agent's discretion." *Journal of European Public Policy* 16 (5):719-37.	delreux_2009
53	Di Lucia, Lorenzo, and Annica Kronsell. 2010. "The willing, the unwilling and the unable - Explaining implementation of the EU biofuels directive." *Journal of European Public Policy* 17 (4):545-63.	dilucia_kronsell_2010
54	Di Paola, Nadia, Rosanna Spanò, Adele Caldarelli, and Roberto Vona. 2018. "Hi-tech start-ups: Legitimacy challenges and funding dynamics." *Technology Analysis & Strategic Management* 30 (3):363-75.	dipaola_et_al_2018
55	Dunlop, Claire A., Martino Maggetti, Claudio M. Radaelli, and Duncan Russel. 2012. "The many uses of regulatory impact assessment: A meta-analysis of EU and UK cases." *Regulation & Governance* 6 (1):23-45.	dunlop_et_al_2012

56	Ebeturk, Irem A., and Oliver Cowart. 2017. “Criminalization of forced marriage in Europe: A qualitative comparative analysis.” *International Journal of Comparative Sociology* 58 (3):169-91.	ebeturk_cowart_2017
57	Eder, Christina. 2010. “A Key to success? Are there conditions for successful ballot votes in the German Länder?” *Politische Vierteljahresschrift* 51 (1):43-67.	eder_2010
58	Elliott, Thomas A., Edwin Amenta, and Neal Caren. 2016. “Recipes for attention: Policy reforms, crises, organizational characteristics, and the newspaper coverage of the LGBT movement, 1969–2009.” *Sociological Forum* 31 (4):926-47.	elliott_et_al_2016
59	Emmenegger, Patrick. 2008. “Religion und Arbeitnehmerschutzgesetzgebung: Eine Fuzzy Set-Analyse.” *Swiss Political Science Review* 14 (1):85-130.	emmenegger_2008
60	Emmenegger, Patrick. 2011. “Job security regulations in Western democracies: A fuzzy set analysis.” *European Journal of Political Research* 50 (3):336-64.	emmenegger_2011a
61	Engeli, Isabelle, and Christine Rothmayr Allison. 2013. “Diverging against all odds? Regulatory paths in embryonic stem cell research across Western Europe.” *Journal of European Public Policy* 20 (3):407-24.	engeli_rothmayrallison_2013
62	Fagerholm, Andreas. 2016. “Social democratic parties and the rise of ecologism: A comparative analysis of Western Europe.” *Comparative European Politics* 14 (5):547-71.	fagerholm_2016
63	Fainshmidt, Stav, Adam Smith, and Orhun Guldiken. 2017. “Orchestrating the flow of human resources: Insights from Spanish soccer clubs.” *Strategic Organization* 15 (4):441-60.	fainshmidt_et_al_2017
64	Fainshmidt, Stav, Adam Smith, and William Q. Judge. 2016. “National competitiveness and Porter’s Diamond Model: The role of MNE penetration and governance quality.” *Global Strategy Journal* 6 (2):81-104.	fainshmidt_et_al_2016
65	Ferguson, Graham, Carol M. Megehee, and Arch G. Woodside. 2017. “Culture, religiosity, and economic configural models explaining tipping-behavior prevalence across nations.” *Tourism Management* 62:218-33.	ferguson_et_al_2017
66	Fink-Hafner, Danica, and Mitja Hafner-Fink. 2009. “The determinants of the success of transitions to democracy.” *Europe-Asia Studies* 61 (9):1603-25.	finkhafner_hafnerfink_2009
67	Fischer, Jörn, André Kaiser, and Ingo Rohlfing. 2006. “The push and pull of ministerial resignations in Germany, 1969-2005.” *West European Politics* 29 (4):709-35.	fischer_et_al_2006
68	Fischer, Manuel. 2014. “Coalition structures and policy change in a consensus democracy.” *Policy Studies Journal* 42 (3):344-66.	fischer_2014
69	Fischer, Manuel. 2015. “Institutions and coalitions in policy processes: A cross-sectoral comparison.” *Journal of Public Policy* 35 (2):245-68.	fischer_2015
70	Fischer, Manuel, and Isabelle Schläpfer. 2017. “Metagovernance and policy forum outputs in Swiss environmental politics.” *Environmental Politics* 26 (5):870-92.	fischer_schlapfer_2017

71 Frazier, M. Lance, Christina Tupper, and Stav Fainshmidt. 2016. "The path(s) to employee trust in direct supervisor in nascent and established relationships: A fuzzy set analysis." *Journal of Organizational Behavior* 37 (7):1023-43. frazier_et_al_2016

72 Freitag, Markus, and Raphaela Schlicht. 2009. "Educational federalism in Germany: Foundations of social inequality in education. *Governance: An International Journal of Policy, Administration, and Institutions* 22 (1):47-72. freitag_schlicht_2009

73 Ganguly, Rajat. 1997. "The move towards disintegration: Explaining ethnosecessionist mobilization in South Asia." *Nationalism and Ethnic Politics* 3 (2):101-30. ganguly_1997

74 Gansemans, Annelien, Deborah Martens, Marijke D'Haese, and Jan Orbie. 2017. "Do labour rights matter for export? A qualitative comparative analysis of pineapple trade to the EU." *Politics and Governance* 5 (4):93-105. gansemans_et_al_2017

75 García-Castro, Roberto, and José Casasola. 2011. "A set-theoretic analysis of the components of family involvement in publicly listed and major unlisted firms." *Journal of Family Business Strategy* 2 (1):15-25. garciacastro_casasola_2011

76 Gherghina, Sergiu, and George Jiglău. 2011. "Explaining ethnic mobilisation in post-communist countries." *Europe-Asia Studies* 63 (1):49-76. gherghina_jiglau_2011

77 Gherghina, Sergiu, and George Jiglău. 2016. "Playing their cards right: Ethnic parties and government coalitions in postcommunist Europe." *Nationalism and Ethnic Politics* 22 (2):220-40. gherghina_jiglau_2016

78 Giugni, Marco, and Alessandro Nai. 2013. "Paths towards consensus: Explaining decision making within the Swiss Global Justice movement." *Swiss Political Science Review* 19 (1):26-40. giugni_nai_2013

79 Giugni, Marco, and Sakura Yamasaki. 2009. "The policy impact of social movements: A replication through qualitative comparative analysis." *Mobilization: An International Quarterly* 14 (4):467-84. giugni_yamasaki_2009

80 Giuliani, Marco. 2016. "Patterns of democracy reconsidered: The ambiguous relationship between corporatism and consensualism." *European Journal of Political Research* 55 (1):22-42. giuliani_2016

81 Gjølberg, Maria. 2009. "The origin of corporate social responsibility: Global forces or national legacies?" *Socio-Economic Review* 7 (4):605-37. gjolberg_2009

82 Glaesser, Judith, and Barry Cooper. 2011. "Selectivity and flexibility in the German secondary school system: A configurational analysis of recent data from the German socio-economic panel." *European Sociological Review* 27 (5):570-85. glaesser_cooper_2011a

83 Glaesser, Judith, and Barry Cooper. 2012. "Educational achievement in selective and comprehensive local education authorities: A configurational analysis." *British Journal of Sociology of Education* 33 (2):223-44. glaesser_cooper_2012a

84 Good, Marin, Simon Hurst, Rahel Willener, and Fritz Sager. 2012. "Die Ausgaben der Schweizer Kantone - Eine Fuzzy Set QCA." *Swiss Political Science Review* 18 (4):452-76. good_et_al_2012

85 Gordin, Jorge P. 2001. "The electoral fate of ethnoregionalist parties in Western Europe: A Boolean test of extant explanations." *Scandinavian Political Studies* 24 (2):149-70. gordin_2001

86 Grant, Don, Alfonso Morales, and Jeffrey J. Sallaz. 2009. "Pathways to meaning: A new approach to studying emotions at work." *American Journal of Sociology* 115 (2):327-64. grant_et_al_2009a

87 Grant, Don, Mary N. Trautner, Liam Downey, and Lisa Thiebaud. 2010. "Bringing the polluters back in: Environmental inequality and the organization of chemical production." *American Sociological Review* 75 (4):479-504. grant_et_al_2010

88 Grassi, Davide. 2004. "La survie des régimes démocratiques: Une AQQC des démocraties de la "Troisième Vague" en Amérique du Sud." *Revue Internationale de Politique Comparée* 11 (1):17-33. grassi_2004

89 Grauvogel, Julia, and Christian von Soest. 2014. "Claims to legitimacy count: Why sanctions fail to instigate democratisation in authoritarian regimes." *European Journal of Political Research* 53 (4):635-53. grauvogel_vonsoest_2014

90 Gross, Martin, and Tim Niendorf. 2017. "Determinanten der Bildung nicht-etablierter Koalitionen in den deutschen Bundesländern, 1990–2016." *Zeitschrift für Vergleichende Politikwissenschaft* 11 (3):365-90. gross_niendorf_2017

91 Guzman-Concha, Cesar. 2015. "Radical social movements in Western Europe: A configurational analysis." *Social Movement Studies* 14 (6):668-91. guzmanconcha_2015

92 Haesebrouck, Tim. 2017a. "NATO burden sharing in Libya: A fuzzy set qualitative comparative analysis." *Journal of Conflict Resolution* 61 (10):2235-61. haesebrouck_2017a

93 Haesebrouck, Tim. 2017b. "EU member state participation in military operations: A configurational comparative analysis." *Cambridge Review of International Affairs* 30 (2-3):137-59. haesebrouck_2017b

94 Hafner-Fink, Mitja, Brina Malnar, and Samo Uhan. 2013. "The national contexts of post-national citizenship." *Czech Sociological Review* 49 (6):867-901. hafnerfink_et_al_2013

95 Hanley, Seán, and Allan Sikk. 2016. "Economy, corruption or floating voters? Explaining the breakthroughs of anti-establishment reform parties in Eastern Europe." *Party Politics* 22 (4):522-33. hanley_sikk_2016

96 Hansen, Janus, and Agnes Allansdottir. 2011. "Assessing the impacts of citizen participation in science governance: Exploring new roads in comparative analysis." *Science and Public Policy* 38 (8):609-17. hansen_allansdottir_2011

97 Hartmann, Christof, and Jörg Kemmerzell. 2010. "Understanding variations in party bans in Africa." *Democratization* 17 (4):642-65. hartmann_kemmerzell_2010

98 Haworth-Hoeppner, Susan. 2000. “The critical shapes of body image: The role of culture and family in the production of eating disorders.” *Journal of Marriage and Family* 62 (1):212-27. haworthhoeppner_2000

99 Hicks, Alexander, Joya Misra, and Tang Nah Ng. 1995. “The programmatic emergence of the Social Security State.” *American Sociological Review* 60 (3):329-49. hicks_et_al_1995

100 Hinterleitner, Markus, Fritz Sager, and Eva Thomann. 2016. “The politics of external approval: Explaining the IMF’s evaluation of austerity programmes.” *European Journal of Political Research* 55 (3):549-67. hinterleitner_et_al_2016

101 Hooijer, Gerda, and Georg Picot. 2015. “European welfare states and migrant poverty: The institutional determinants of disadvantage.” *Comparative Political Studies* 48 (14):1879-904. hooijer_picot_2015

102 Hörisch, Felix. 2013. “Fiscal policy in hard times: A fuzzy-set QCA of fiscal policy reactions to the financial crisis.” *Zeitschrift für Vergleichende Politikwissenschaft* 7 (2):117-41. horisch_2013

103 Hotho, Jasper J. 2014. “From typology to taxonomy: A configurational analysis of national business systems and their explanatory power.” *Organization Studies* 35 (5):671-702. hotho_2014

104 Hussain, Muzammil M., and Philip N. Howard. 2013. “What best explains successful protest cascades? ICTs and the fuzzy causes of the Arab Spring.” *International Studies Review* 15 (1):48-66. hussain_howard_2013

105 Iannotta, Michela, Mauro Gatti, and Morten Huse. 2016. “Institutional complementarities and gender diversity on boards: A configurational approach.” *Corporate Governance: An International Review* 24 (4):406-27. iannotta_et_al_2016

106 Ide, Tobias. 2018. “Does environmental peacemaking between states work? Insights on cooperative environmental agreements and reconciliation in international rivalries.” *Journal of Peace Research* 55 (3): 351-65. ide_2018

107 Ignatow, Gabe. 2011. “What has globalization done to developing countries’ public libraries?” *International Sociology* 26 (6):746-68. ignatow_2011

108 Ingrams, Alex. 2017. “The legal-normative conditions of police transparency: A configurational approach to open data adoption using qualitative comparative analysis.” *Public Administration* 95 (2):527-45. ingrams_2017

109 Iseke, Anja, Birgit Kocks, Martin R. Schneider, and Conrad Schulze-Bentrop. 2015. “Cross-cutting organizational and demographic divides and the performance of research and development teams: Two wrongs can make a right.” *R&D Management* 45 (1):23-40. iseke_et_al_2015

110	Ishiyama, John, and Anna Batta. 2012. "The emergence of dominant political party systems in unrecognized states." *Communist and Post-Communist Studies* 45 (1–2):123-30.	ishiyama_batta_2012
111	Jano, Dorian. 2016. "Compliance with EU legislation in the pre-accession countries of South East Europe (2005–2011): A fuzzy-set qualitative comparative analysis." *Journal of European Integration* 38 (1):1-22.	jano_2016
112	Jennings, Will, Martin Lodge, and Matt Ryan. 2018. "Comparing blunders in government." *European Journal of Political Research* 57 (1): 238-58.	jennings_et_al_2018
113	Judge, William Q., Stav Fainshmidt, and J. Lee Brown Iii. 2014. "Which model of capitalism best delivers both wealth and equality?." *Journal of International Business Studies* 45 (4):363-86.	judge_et_al_2014
114	Judge, William Q., Helen W. Hu, Jonas Gabrielsson, Till Talaulicar, Michael A. Witt, Alessandro Zattoni, ..., and Bruce Kibler. 2015. "Configurations of capacity for change in entrepreneurial threshold firms: Imprinting and strategic choice perspectives." *Journal of Management Studies* 52 (4):506-30.	judge_et_al_2015
115	Kammermann, Lorenz. 2018. "Factors driving the promotion of hydroelectricity: A qualitative comparative analysis." *Review of Policy Research* 35 (2):213-37.	kammermann_2018
116	Karatzas, Antonios, Mark Johnson, and Marko Bastl. 2016. "Relationship determinants of performance in service triads: A configurational approach." *Journal of Supply Chain Management* 52 (3):28-47.	karatzas_et_al_2016
117	Karlas, Jan. 2012. "National parliamentary control of EU affairs: Institutional design after enlargement." *West European Politics* 35 (5):1095-113.	karlas_2012
118	Karlas, Jan. 2017. "States, coalitions, and the legalization of the global climate regime: Negotiations on the post-2020 architecture." *Environmental Politics* 26 (5): 825-46.	karlas_2017
119	Keudel-Kaiser, Dorothea. 2016. "Party system factors and the formation of minority governments in central and eastern Europe." *Zeitschrift für Vergleichende Politikwissenschaft* 10 (3):341-69.	keudelkaiser_2016
120	Klüver, Heike. 2010. "Europeanization of lobbying activities: When national interest groups spill over to the European level." *Journal of European Integration* 32 (2):175-91.	kluver_2010
121	Kopraleva, Iva, and Maarten P. Vink. 2015. "EU sanctions in response to intra-state conflicts: A comparative approach." *European Foreign Affairs Review* 20 (3):315-36.	kopraleva_vink_2015
122	Kosmol, Tobias, Felix Reimann, and Lutz Kaufmann. 2018. "Co-alignment of supplier quality management practices and cognitive maps - a neo-configurational perspective." *Journal of Purchasing and Supply Management* 24 (1):1-20.	kosmol_et_al_2018
123	Krook, Mona L. 2010. "Women's representation in parliament: A qualitative comparative analysis." *Political Studies* 58 (5):886-908.	krook_2010

124	Kühn, David, Aurel Croissant, Jil Kamerling, Hans Lueders, and André Strecker. 2017. "Conditions of civilian control in new democracies: An empirical analysis of 28 'Third Wave' democracies." *European Political Science Review* 9 (3):425-47.	kuhn_et_al_2017
125	Kühn, David, and Harold Trinkunas. 2017. "Conditions of military contestation in populist Latin America." *Democratization* 24 (5):859-80.	kuhn_trinkunas_2017
126	Li, Yanwei, Joop Koppenjan, and Stefan Verweij. 2016. "Governing environmental conflicts in China: Under what conditions do local governments compromise?" *Public Administration* 94 (3):806-22.	li_et_al_2016
127	Lilliefeldt, Emelie. 2012. "Party and gender in Western Europe revisited: A fuzzy-set Qualitative Comparative Analysis of gender-balanced parliamentary parties." *Party Politics* 18 (2):193-214.	lilliefeldt_2012
128	Linder, Wolf. 2010. "On the merits of decentralization in young democracies." *Publius: The Journal of Federalism* 40 (1):1-30.	linder_2010
129	Lindemann, Stefan, and Andreas Wimmer. 2018. "Repression and refuge: Why only some politically excluded ethnic groups rebel." *Journal of Peace Research* 55 (3):305-19.	lindemann_wimmer_2018
130	Lowik, Sandor, Jeroen Kraaijenbrink, and Aard Groen. 2016. "The team absorptive capacity triad: A configurational study of individual, enabling, and motivating factors." *Journal of Knowledge Management* 20 (5):1083-103.	lowik_et_al_2016
131	Maatsch, Aleksandra. 2014. "Are we all austerians now? An analysis of national parliamentary parties' positioning on anti-crisis measures in the Eurozone." *Journal of European Public Policy* 21 (1):96-115.	maatsch_2014
132	Maggetti, Martino. 2009. "The role of independent regulatory agencies in policy-making: A comparative analysis." *Journal of European Public Policy* 16 (3):450-70.	maggetti_2009
133	Maggetti, Martino, and Fabrizio Gilardi. 2016. "Problems (and solutions) in the measurement of policy diffusion mechanisms." *Journal of Public Policy* 36 (1):87-107.	maggetti_gilardi_2016
134	Mantilla, Luis F. 2010. "Mobilizing religion for democracy: Explaining catholic church support for democratization in South America." *Politics and Religion* 3 (3):553-79.	mantilla_2010
135	Marcos-Marne, Hugo. 2016. "Autonomist and secessionist parties in post-communist democracies. Structural and institutional factors in the study of a dynamic phenomenon." *National Identities* 18 (4):379-96.	marcosmarne_2016
136	Marques, Paulo, and Isabel Salavisa. 2017. "Young people and dualization in Europe: A fuzzy set analysis." *Socio-Economic Review* 15 (1):135-60.	marques_salavisa_2017
137	Martí I Puig, Salvador. 2010. "Les raisons de l'existence et du succès des partis ethniques en Amérique Latine. Les cas de la Bolivie, de l'Équateur, du Guatemala, du Mexique, du Nicaragua et du Pérou." *Revue Internationale de Politique Comparée* 17 (2):143-65.	marti_2010

138	Martín-de Castro, Gregorio, Miriam Delgado-Verde, Javier Amores-Salvadó, and José E. Navas-López. 2013. "Linking human, technological, and relational assets to technological innovation: Exploring a new approach." *Knowledge Management Research & Practice* 11 (2):123-32.	martindecastro_et_al_2013
139	Marx, Axel. 2008. "Limits to non-state market regulation: A Qualitative Comparative Analysis of the international sport footwear industry and the Fair Labor Association." *Regulation & Governance* 2 (2):253-73.	marx_2008
140	Mello, Patrick A. 2012. "Parliamentary peace or partisan politics? Democracies' participation in the Iraq War." *Journal of International Relations and Development* 15 (3):420-53.	mello_2012
141	Metelits, Claire M. 2009. "The consequences of rivalry: Explaining insurgent violence using fuzzy sets." *Political Research Quarterly* 62 (4):673-84.	metelits_2009
142	Mihaila, Roxana. 2012. "Post-accession effects of conditionality: New member states and the implementation of the EU competition policy." *Romanian Journal of Political Science* 12 (1):102-33.	mihaila_2012
143	Misangyi, Vilmos F. 2016. "Institutional complexity and the meaning of loose coupling: Connecting institutional sayings and (not) doings." *Strategic Organization* 14 (4):407-40.	misangyi_2016
144	Misra, Joya. 2003. "Women as agents in welfare state development: A cross-national analysis of family allowance adoption." *Socio-Economic Review* 1 (2):185-214.	misra_2003
145	Mochtak, Michal. 2016. "Explaining electoral violence in Serbia: fsQCA analysis of contentious behavior in the electoral arena." *Democracy and Security* 12 (4):278-308.	mochtak_2016
146	Møller, Jørgen, and Svend-Erik Skaaning. 2009. "The three worlds of post-communism: Revisiting deep and proximate explanations." *Democratization* 16 (2):298-322.	moller_skaaning_2009
147	Moraski, Bryon J. 2013. "Constructing courts after communism: Reevaluating the effect of electoral uncertainty." *Communist and Post-Communist Studies* 46 (4):433-43.	moraski_2013
148	Nelson, Jennifer L. 2017. "Pathways to green(er) pastures: Reward bundles, human capital, and turnover decisions in a semi-profession." *Qualitative Sociology* 40 (1):23-57.	nelson_2017
149	Nieto Morales, Fernando, Rafael Wittek, and Liesbet Heyse. 2015. "Organizational pathways to compliant reform implementation: Evidence from Mexican civil service reform." *Public Administration* 93 (3):646-62.	nietomorales_et_al_2015
150	Ordanini, Andrea, and Paul P. Maglio. 2009. "Market orientation, internal process, and external network: A qualitative comparative analysis of key decisional alternatives in the new service development." *Decision Sciences* 40 (3):601-25.	ordanini_maglio_2009
151	Palm, Trineke. 2013. "Embedded in social cleavages: An explanation of the variation in timing of women's suffrage." *Scandinavian Political Studies* 36 (1):1-22.	palm_2013

152 Pennings, Paul. 2003. “Beyond dichotomous explanations: Explaining constitutional control of the executive with fuzzy-sets.” *European Journal of Political Research* 42 (4):541-67. pennings_2003

153 Pérez Durán, Ixchel, and Jorge Rodríguez Menés. 2017. “Explaining accountability for public policies: An fsQCA analysis of health policy in Spain.” *European Political Science Review* 9 (3):329-50. perezduran_rodriguezmenes_2017

154 Perry, Ashley M., and Mark J. Schafer. 2014. “Resilience in Louisiana FEMA parks: A person-centered, fuzzy-set analysis.” *Sociological Spectrum* 34 (1):39-60. perry_schafer_2014

155 Pogrebinschi, Thamy, and Matt Ryan. 2018. “Moving beyond input legitimacy: When do democratic innovations affect policy making?”. *European Journal of Political Research* 57 (1):135-52. pogrebinschi_ryan_2018

156 Portes, Alejandro, and Lori D. Smith. 2008. “Institutions and development in Latin America: A comparative analysis.” *Studies in Comparative International Development* 43 (2):101-28. portes_smith_2008

157 Portes, Alejandro, and Lori D. Smith. 2010. “Institutions and national development in Latin America: A comparative study.” *Socio-Economic Review* 8 (4):585-621. portes_smith_2010

158 Pullum, Amanda. 2016. “Social movements, strategic choice, and recourse to the polls.” *Mobilization: An International Quarterly* 21 (2):177-92. pullum_2016

159 Qin, Hua, Elizabeth Bent, Caroline Brock, Yassine Dguidegue, Elizabeth Achuff, Meghan Hatcher, and Ojetunde Ojewola. 2018. “Fifteen years after the Bellingham ISSRM: An empirical evaluation of Frederick Buttel’s differentiating criteria for environmental and resource sociology.” *Rural Sociology* 83 (1):6-23. qin_et_al_2018

160 Redding, Kent, and Jocelyn S. Viterna. 1999. “Political demands, political opportunities: Explaining the differential success of left-libertarian parties.” *Social Forces* 78 (2):491-510. redding_viterna_1999

161 Reimann, Felix, Tobias Kosmol, and Lutz Kaufmann. 2017. “Responses to supplier-induced disruptions: A fuzzy-set analysis.” *Journal of Supply Chain Management* 53 (4):37-66. reimann_et_al_2017

162 Reynaert, Vicky. 2011. “Explaining EU aid allocation in the Mediterranean: A fuzzy-set Qualitative Comparative Analysis.” *Mediterranean Politics* 16 (3):405-26. reynaert_2011

163 Rihoux, Benoît. 2006. “Governmental participation and the organizational adaptation of green parties: On access, slack, overload and distress.” *European Journal of Political Research* 45 (S1):69-98. rihoux_2006

164 Ryan, Matt, and Graham Smith. 2012. “Towards a comparative analysis of democratic innovations. Lessons from a small-N fsQCA of participatory budgeting.” *Revista Internacional de Sociología* 70 (S2):89-120. ryan_smith_2012

165 Sager, Fritz. 2004a. "Metropolitan institutions and policy coordination: The integration of land use and transport policies in Swiss urban areas." *Governance: An International Journal of Policy, Administration, and Institutions* 18 (2):227-56. sager_2004a

166 Sager, Fritz. 2004b. "Institutions métropolitaines et coordination des politiques publiques: Une AQQC des arrangements politico-administratifs d'articulation entre urbanisme et transports en Europe." *Revue Internationale de Politique Comparée* 11 (1):67-84. sager_2004b

167 Sager, Fritz. 2006. "Policy coordination in the European metropolis: A meta-analysis." *West European Politics* 29 (3):433-60. sager_2006

168 Sager, Fritz. 2008. "Institutionelle Bedingungen kollektiver Handlungsfähigkeit im urbanen Raum: Eine QCA von siebzehn europäischen Entscheidungsfällen." *Zeitschrift für Vergleichende Politikwissenschaft* 2 (1):44-69. sager_2008

169 Sager, Fritz. 2010. "Institutional preconditions for the collective capacity to act in urban areas: A QCA of seventeen European case studies." *Zeitschrift für Vergleichende Politikwissenschaft* 4:55-77. sager_2010

170 Sager, Fritz, and Markus Hinterleitner. 2016. "How do credit rating agencies rate? An implementation perspective on the assessment of austerity programs during the European debt crisis." *Politics & Policy* 44 (4):783-815. sager_hinterleitner_2016

171 Sager, Fritz, and Eva Thomann. 2017. "Multiple streams in member state implementation: Politics, problem construction and policy paths in Swiss asylum policy." *Journal of Public Policy* 37 (3):287-314. sager_thomann_2017

172 Samford, Steven. 2010. "Averting "disruption and reversal": Reassessing the logic of rapid trade reform in Latin America." *Politics & Society* 38 (3):373-407. samford_2010

173 Schimmelfennig, Frank, Berthold Rittberger, Alexander Bürgin, and Guido Schwellnus. 2006. "Conditions for EU constitutionalization: A Qualitative Comparative Analysis." *Journal of European Public Policy* 13 (8):1168-89. schimmelfennig_et_al_2006

174 Schneider, Carsten Q., and Kristin Makszin. 2014. "Forms of welfare capitalism and education-based participatory inequality." *Socio-Economic Review* 12 (2):437-62. schneider_makszin_2014

175 Schneider, Carsten Q., and Seraphine F. März. 2017. "Legitimation, cooptation, and repression and the survival of electoral autocracies." *Zeitschrift für Vergleichende Politikwissenschaft* 11 (2):213-35. schneider_marz_2017

176 Schneider, Martin R., Conrad Schulze-Bentrop, and Mihai Paunescu. 2010. "Mapping the institutional capital of high-tech firms: A fuzzy-set analysis of capitalist variety and export performance." *Journal of International Business Studies* 41 (2):246-66. schneider_et_al_2010

177 Schoon, Eric W. 2014. "The asymmetry of legitimacy: Analyzing the legitimation of violence in 30 cases of insurgent revolution." *Social Forces* 93 (2):779-801. schoon_2014

178 Schulte, Felix. 2017. "Two birds, one stone – War-to-democracy processes after ethnic conflicts." *S&F Sicherheit und Frieden* 35 (1):34-45. schulte_2017

179 Schwarz, Oliver. 2016. "Two steps forward one step back: What shapes the process of EU enlargement in south-eastern Europe?" *Journal of European Integration* 38 (7):757-73. schwarz_2016

180 Sidki Darendeli, Izzet, and T. L. Hill. 2016. "Uncovering the complex relationships between political risk and MNE firm legitimacy: Insights from Libya." *Journal of International Business Studies* 47 (1):68-92. sidkidarendeli_hill_2016

181 Smilde, David. 2005. "A qualitative comparative analysis of conversion to Venezuelan Evangelicalism: How networks matter." *American Journal of Sociology* 111 (3):757-96. smilde_2005

182 Stevens, Alex. 2016. "Configurations of corruption: A cross-national qualitative comparative analysis of levels of perceived corruption." *International Journal of Comparative Sociology* 57 (4):183-206. stevens_2016b

183 Stiller, Sabina. 2017. "The interplay of actor-related strategies and political context: A fuzzy-set QCA analysis of structural reforms in continental welfare states." *Journal of European Public Policy* 24 (1):81-99. stiller_2017

184 Stoiber, Michael, and Annette E. Töller. 2016. "Studying the causes of privatization of hospital order treatment in Germany. A Qualitative Comparative Analysis of the German Länder." *Zeitschrift für Vergleichende Politikwissenschaft* 10 (1):9-36. stoiber_toller_2016

185 Strandberg, Kim. 2008. "Online electoral competition in different settings - A comparative meta-analysis of the research on party websites and online electoral competition." *Party Politics* 14 (2):223-44. strandberg_2008

186 Striebing, Clemens. 2017. "Professionalization and voluntary transparency practices in nonprofit organizations." *Nonprofit Management and Leadership* 28 (1):65-83. striebing_2017

187 Takahashi, Takuya, and Masao Nakamura. 2005. "Bureaucratization of environmental management and corporate greening: An empirical analysis of large manufacturing firms in Japan." *Corporate Social Responsibility and Environmental Management* 12 (4):210-219. takahashi_nakamura_2005

188 Tan, Chee-Wee, Izak Benbasat, and Ronald T. Cenfetelli. 2016. "An exploratory study of the formation and impact of electronic service failures." *MIS Quarterly* 40 (1):1-29. tan_et_al_2016

189 Thiem, Alrik. 2011. "Conditions of intergovernmental armaments cooperation in Western Europe, 1996-2006." *European Political Science Review* 3 (1):1-33. thiem_2011

190 Thomann, Eva. 2015a. "Customizing Europe: Transposition as bottom-up implementation." *Journal of European Public Policy* 22 (10):1368-87. thomann_2015a

191 Thomann, Eva. 2015b. "Is output performance all about the resources? A fuzzy-set Qualitative Comparative Analysis of street-level bureaucrats in Switzerland." *Public Administration* 93 (1):177-94. thomann_2015b

192	Timmer, Stephane, and Lutz Kaufmann. 2017. "Conflict minerals traceability - a fuzzy set analysis." *International Journal of Physical Distribution & Logistics Management* 47 (5):344-67.	timmer_kaufmann_2017
193	Tobin, Paul. 2017. "Leaders and laggards: Climate policy ambition in developed states." *Global Environmental Politics* 17 (4): 28-47.	tobin_2017
194	Toomla, Raul. 2016. "Charting informal engagement between de facto states: A quantitative analysis." *Space and Polity* 20 (3):330-45.	toomla_2016
195	van der Heijden, Jeroen. 2015. "The role of government in voluntary environmental programmes: A fuzzy set Qualitative Comparative Analysis." *Public Administration* 93 (3):576-92.	vanderheijden_2015
196	van der Maat, Eelco. 2011. "Sleeping hegemons: Third-party intervention following territorial integrity transgressions." *Journal of Peace Research* 48 (2):201-15.	vandermaat_2011
197	Vandecasteele, Bruno, Fabienne Bossuyt, and Jan Orbie. 2015. "A fuzzy-set Qualitative Comparative Analysis of the Hungarian, Polish and Lithuanian presidencies and European Union eastern partnership policies." *European Politics and Society* 16 (4):556-80.	vandecasteele_et_al_2015
198	Varone, Frédéric, Christine Rothmayr, and Eric Montpetit. 2006. "Regulating biomedicine in Europe and North America: A Qualitative Comparative Analysis." *European Journal of Political Research* 45 (2):317-43.	varone_et_al_2006
199	Vergne, Jean-Philippe, and Colette Depeyre. 2016. "How do firms adapt? A fuzzy-set analysis of the role of cognition and capabilities in U.S. defense firms' responses to 9/11." *Academy of Management Journal* 59 (5):1653-80.	vergne_depeyre_2016
200	Verweij, Stefan. 2015a. "Achieving satisfaction when implementing PPP transportation infrastructure projects: A qualitative comparative analysis of the AA15 highway DBFM project." *International Journal of Project Management* 33 (1):189-200.	verweij_2015a
201	Verweij, Stefan. 2015b. "Producing satisfactory outcomes in the implementation phase of PPP infrastructure projects: A fuzzy set qualitative comparative analysis of 27 road constructions in the Netherlands." *International Journal of Project Management* 33 (8):1877-87.	verweij_2015b
202	Verweij, Stefan, Erik-Hans Klijn, Jurian Edelenbos, and Arwin Van Buuren. 2013. "What makes governance networks work? A fuzzy set Qualitative Comparative Analysis of 14 Dutch spatial planning projects." *Public Administration* 91 (4):1035-55.	verweij_et_al_2013
203	Veugelers, John, and André Magnan. 2005. "Conditions of far-right strength in contemporary Western Europe: An application of Kitschelt's theory." *European Journal of Political Research* 44 (6):837-60.	veugelers_magnan_2005
204	Vis, Barbara. 2009. "Governments and unpopular social policy reform: Biting the bullet or steering clear?" *European Journal of Political Research* 48 (1):31-57.	vis_2009

205	Vis, Barbara. 2011. "Under which conditions does spending on active labor market policies increase? An fsQCA analysis of 53 governments between 1985 and 2003." *European Political Science Review* 3 (2):229-52.	vis_2011
206	von Kulessa, Alexander, and Georg Wenzelburger. 2015. "Starker Steuerwettbewerb – starke Reformen? Ein neuer Blick auf Unternehmenssteuerreformen in 15 EU-Staaten (1998-2011)." *Swiss Political Science Review* 21 (2):302-32.	vonkulessa_wenzelburger_2015
207	Weinberg, Adam S. 1997. "Local organizing for environmental conflict." *Organization & Environment* 10 (2):194-216.	weinberg_1997
208	Wickham-Crowley, Timothy P. 1991. "A qualitative comparative approach to Latin American revolutions." *International Journal of Comparative Sociology* 32 (1-2):82-109.	wickhamcrowley_1991
209	Wollebæk, Dag. 2010. "Volatility and growth in populations of rural associations." *Rural Sociology* 75: 144-66.	wollebaek_2010
210	Yamasaki, Sakura. 2009. "A Boolean analysis of movement impact on nuclear energy policy." *Mobilization: An International Journal* 14 (4):485-504.	yamasaki_2009
211	Yan, Huiqi, Jeroen van der Heijden, and Benjamin van Rooij. 2017. "Symmetric and asymmetric motivations for compliance and violation: A crisp set Qualitative Comparative Analysis of Chinese farmers." *Regulation & Governance* 11 (1):64-80.	yan_et_al_2017
212	Young, Kevin L., and Sung Ho Park. 2013. "Regulatory opportunism: Cross-national patterns in national banking regulatory responses following the global financial crisis." *Public Administration* 91 (3):561-81.	young_park_2013
213	Zeng, Jinghan. 2013. "What matters most in selecting top Chinese leaders? A Qualitative Comparative Analysis." *Journal of Chinese Political Science* 18 (3):223-39.	zeng_2013
214	Zimmermann, Katharina. 2016. "Local responses to the European social fund: A cross-city comparison of usage and change." *Journal of Common Market Studies* 54 (6):1465-84.	zimmermann_2016
215	Zupan, Blaž, Aleš Pustovrh, and Stanka S. Cankar. 2017. "Does decentralized governance lead to less scientific output? A fuzzy set analysis of fiscal decentralization and determinants of national innovation capacity." *Lex Localis: Journal of Local Self-Government* 15 (3):625-45.	zupan_et_al_2017

Table A1: List of included QCA studies
