# Supplementary material for: Algorithmic bias in social research: A meta-analysis
Source: PLoS One. 2020 Jun 8;15(6):e0233625. doi: 10.1371/journal.pone.0233625 (PMC7279593; doi:10.1371/journal.pone.0233625)
Supplement: S2 Appendix — (PDF) [file pone.0233625.s002.pdf]

Appendix A2: Data Collection Strategy

Our data collection strategy is visualized in the flow diagram shown in Figure 1. The initial database that contains the 215 articles listed in Appendix A1 is a private database of 1'221 QCA-related (as of April 2018), peer-reviewed journal articles that have appeared since April 1984. This database also underlies the COMPASSS bibliography at http://compasss.org/bibliography/. As a method-specific database, it is not only more inclusive than the Web of Science or Scopus, but also more accurate because each entry has already been manually verified before inclusion.[1]

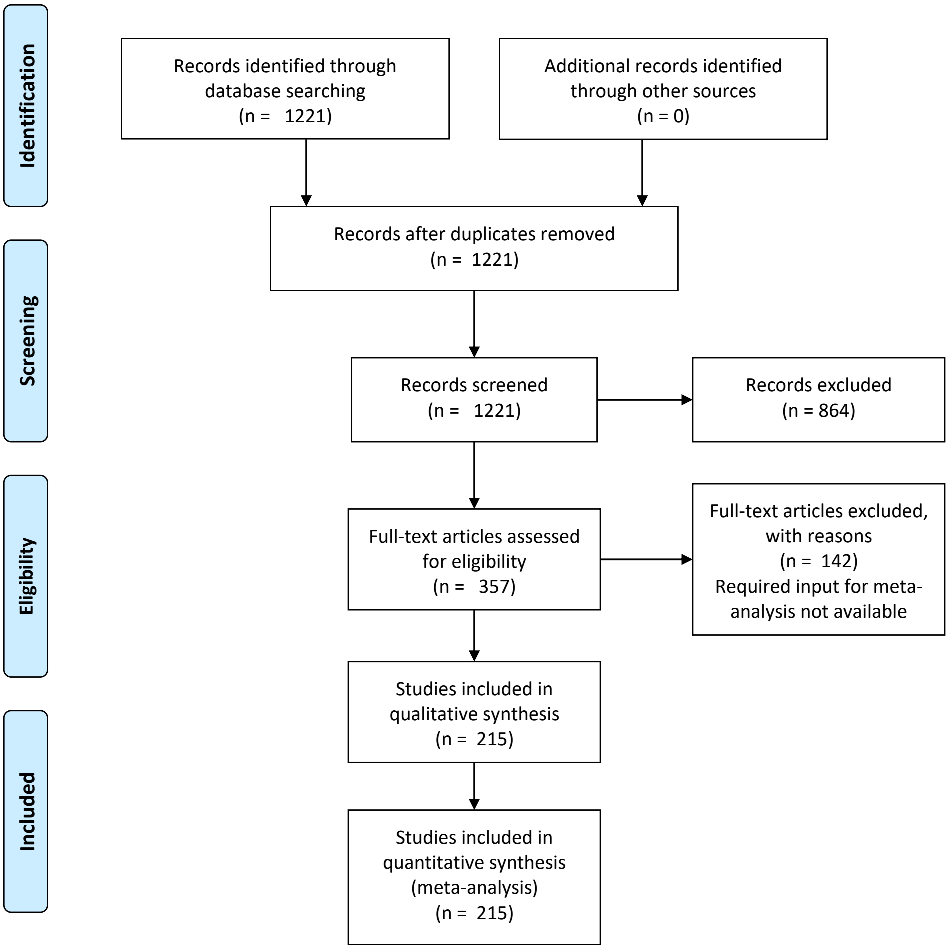

Figure 1: PRISMA flow-diagram of the search strategy and study selection process

We then identified those articles that mainly addressed an empirical research question

[1]For example, Scopus lists the mini-symposium on QCA that has appeared in *Political Research Quarterly*, volume 66, issue 1, as a single publication with 21 co-authors, but it actually is a collection of nine individual articles with varying co-author constellations.

by applying QCA, and that were published in a journal listed in the categories "Political Science", "Management" or "Sociology" of Clarivate Analytics' Journal Citation Reports' (JCR), i.e. a journal that had an impact factor assigned by Clarivate Analytics (previously Thomson Reuters) under the respective category, with a publication date between April 1984 and April 2018. As the publication date, we used the date of full print-issue inclusion, not the online publication date. This resulted in the exclusion of 864 articles and left us with 357 applied/empirical QCA articles.[2]

We decided to focus on studies with a JCR impact factor because for only a small fraction of empirical articles was suitable replication material available in the first place. So as to limit the number of publications for which we would try to obtain this material, we decided to use the JCR impact factor because publications that have appeared in JCR-listed journals are usually rated higher in quality by scientists than those without this metric. In other words, we should have the highest-quality QCA studies, at least if one follows the usual use of Clarivate Analytics' JCR 2-year impact factor in scientific and science-policy communities. However, we grant that there exist competing logics, insofar as it is often not the best research in the sense of the most conscientious work that appears in high impact factor journals, but work that presents strong findings, findings that overwhelmingly support the authors' hypotheses, etc.

We then tried to obtain the data that were used in these 357 articles from the main text or supplementary material. If that proved impossible, we codified the truth table, if available, to mimic the minimization process. For articles from political science and management, we also contacted the study's authors three times (initial request plus two reminders) to ask whether they would grant us access to their data so as to avoid the possibility that data sets, which almost always were available only in non-importable format (tables in doc or pdf documents), contained typos. The text of the initial request e-mail is provided below:

Dear Dr. $Name$,

We are a team of researchers working on a meta-scientific project involving the use of Qualitative Comparative Analysis (QCA) for addressing empirical research questions. To this end, we are currently contacting the corresponding authors of $number of category articles$ QCA articles published in journals listed in Clarivate Analytics' Journal Citation Reports under the category $category$. In this connection, we would like to ask

[2]For example, articles dealing with methodological questions or those whose main objective it was to introduce the method of QCA to a specific research community were not classified as applied/empirical.

you whether it would be possible that you send us the set of calibrated data you have used for the QCA analysis in your article

$Reference$

for purposes of replication, ideally as a comma-separated value (csv) file? We would only need the data set that was ultimately analyzed in your QCA software, not the raw data or any intermediate data.

If you would like to receive more details about our project, we are happy to answer all questions you may have. Also, if you would like us to keep you updated about our project, please let us know.

We thank you very much for your cooperation and assistance.

(If you are the corresponding author of several QCA articles, you will receive this data request e-mail for each article separately. We apologize in advance for this technical necessity.)

This approach regularly resulted in considerable reconstruction efforts because authors did not remember which variables they included, or they used different labels from those used in the article, or dropped cases that were still included in the data set etc. Because of the enormous consumption of human resources these reconstruction efforts required, and the high rate of failure in reconstructing the exact data, we decided not to do the same for sociology and only include those studies where we did not have to contact authors for replication material, but found this material inside the main text or in supplementary resources. Overall, we could not obtain replication material that would have allowed us to re-run the original analysis for 142 articles.

The 215 empirical articles listed in Appendix A1 are those for which we had sufficient accurate information in the end to fully re-run the QCA analysis in the same way the authors originally did. Specifically, we recorded the following information: (1) whether we used the actual data or the truth table in the reanalysis (important because that affects criterion (4)), (2) the solution type of QCA used (parsimonious, intermediate, complex), (3) the consistency cut-off, (4) the frequency cut-off, and (5) the number of models reported.
