# Supplementary material for: Algorithmic bias in social research: A meta-analysis
Source: PLoS One. 2020 Jun 8;15(6):e0233625. doi: 10.1371/journal.pone.0233625 (PMC7279593; doi:10.1371/journal.pone.0233625)
Supplement: Attachment — Submitted filename: response_letter.pdf [file pone.0233625.s005.pdf]

Response to Reviewers

We thank the two reviewers for their feedback on the first revision of our manuscript. In this second response letter, we answer to each comment of the two reviewers, explaining how we have addressed the issues raised, or why we did not implement a suggested change. When we did not implement a reviewer's suggested change, we have made every effort to justify this decision, which we have reached only after careful consideration, in sufficient detail.

REVIEWER 1

- **Comment 1**: In their response to my first comment, the authors say "The fact that multiple explanations often fit a set of data is not unique to QCA, but also exists in regression analysis and experiments (e.g., Eberhardt 2013)". That's intriguing. I didn't know about that, and I think most researchers using regressions and experiments don't know either. It would be very helpful if the author could add a footnote in the main text to explain the intuition behind this claim.

  ***Response*:** We thank Reviewer 1 for this suggestion. We are surprised that it seems not to be common knowledge that there are often multiple explanations / theories / models fitting the same set of data / evidence. For example, in Coincidence Analysis (CNA), the method closely related to QCA, it is even, in principle, NEVER possible to distinguish between a causal-chain structure and a common-cause structure, irrespective of the amount of data available (Baumgartner 2008). In courts of law, the well-known principle of *in dubio pro reo* says nothing more than that there are two arguments, that of the plaintiff and that of the defendant, but the evidence neither allows the judge to conclude that the plaintiff has a clear case nor that the defendant can be cleared without doubt.

  As much as we would like to say more about these issues, footnotes are, unfortunately, not allowed in PLOS ONE articles, and a separate paragraph or even section in the main text would not really fit anywhere without significant changes and additions to the text that may distract the reader from its main argument. However, we take the comment of Reviewer 1 as motivation that a stand-alone paper on this topic may be worth consideration.

REVIEWER 2

The revision has addressed most of the points raised by myself and the other reviewer, and the paper is much improved. There are just three points which the authors have not addressed satisfactorily.

- **Comment 1**: The information on the number of eligible papers does need to be in the main body of the text (my previous comment 2). Contrary to what the authors say, this is not related to reviewer 1's comment 3 but it is a separate point: The authors have identified a population of 360, and they have been able to analyse 215 out of these 360 papers. The

reasons they give for not analysing them all are perfectly acceptable, but they claim that "our analysis suggests that every third QCA solution presented in a management, political science or sociology journal has oversold its solution simply because the algorithm in the chosen QCA software operated under an objective function that is suitable for a specific purpose in electrical engineering applications, but not for causal inference" (p.17). Readers should be aware that this claim concerning "every third QCA solution" rests on a sample roughly 60% the size of the population identified by the authors.

***Response*:** We have included this piece of information now in the section "Data" on page 14.

- **Comment 2**: Adding a reference has not really addressed the point concerning reproducibility (my previous comment 5). There would have needed to be evidence that authors try and fail to replicate findings based on QCA in the first place (I am not aware that there are many attempts to do this), before analysing whether algorithmic bias is to blame. Given that the algorithmic bias discussed in this paper arises only in the context of QCA, it is hard to see how it could contribute to irreproducible findings elsewhere.

***Response*:** There seems to be a (continued) misunderstanding. Algorithmic bias is a general phenomenon prevalent in many research areas (see references 6-8 in the manuscript, and our reply to comment 3 below), whereby a correct result ("correct" in relation to the search target of the analysis) is distorted by the procedures of an algorithm. QCA is also affected by algorithmic bias, as we demonstrate in our manuscript, but the specific type of algorithmic bias, whereby certain irredundant sums are systematically suppressed, is, of course, specific to QCA. For other methods, the exact type of algorithmic bias may be a different one.

Reviewer 2 says s/he is unaware of attempts to replicate QCA findings. In contrast, we know of often-cited publications that have hinted at the possibility that many QCA results may be irreproducible: Thiem and Dușa (2013) have already shown that the three QCA software packages *Tosmana*, *fs/QCA* and *QCA* produce very different results even under the same procedural constraints. These authors surmise that this must be due to hidden differences in the implemented algorithms. Another study that identifies such problems in the field of evaluation research is Thiem (2014). And Baumgartner and Thiem (2017), which has become the second most-cited publication in *Sociological Methods & Research* of the past 3 years (see https://journals.sagepub.com/home/smr) have demonstrated for 28 articles from sociology that some QCA software packages fail to consistently produce rival models that would have been directly identifiable from the data. Whether these studies represent "many attempts" is surely debatable, but we believe that even ***one*** solidly argued indication of (serious) problems of irreproducibility in QCA should suffice to warrant further investigation on the exact nature and scope of this problem. We provide such an investigation in our manuscript with an unprecedentedly large data set.

- **Comment 3**: The title is misleading (my previous comment 6), contrary to the authors' claim. Algorithmic bias is an important problem, but it is one specific to QCA which in turn is one of many methods employed within social science. The fact that other problems "may" exist in the social sciences does not change this

Response**:** We believe that the current title has the potential to attract the attention of a larger community of social scientists for whom our general insights are relevant. If we changed it to, for example, “Algorithmic bias in configurational comparative research” or, even narrower, “Algorithmic bias in Qualitative Comparative Analysis”, the problem that algorithmic bias is not unique to configurational methods but also other research fields would perhaps not be noticed beyond the relatively small community of researchers working on or with configurational methods or even just QCA.

The available evidence seems to support this intuition due to the availability of numerous complex statistical packages and the unfamiliarity of users with the algorithms used in many methods (Bergmann, Ludbrook & Spooren 2000). For example, Günther and Höfler (2006) show how results differ when computing tetrachorical correlations with two alternative statistical packages, Stata and Mplus. The reason behind the substantial discrepancies in the coefficients was the use of different algorithms by the two software packages. Given that both programs have been used extensively across many scientific disciplines, the implications are, potentially, highly consequential. And Eklund et al (2016) analyze differences in the output generated by three magnetic-resonance-imaging software suites as compared to a baseline procedure, only to find “the need of validating the statistical methods being used in the field of neuroimaging” because the implemented algorithmic procedures prove unsuitable for a variety of scenarios.

REFERENCES

Baumgartner, M. (2008). The causal chain problem. *Erkenntnis* 69 (2):201-26.

Baumgartner, M., & Thiem, A. (2017). Model ambiguities in configurational comparative research. *Sociological Methods & Research* 46 (4):954-87.

Bergmann, R., Ludbrook, J., & Spooren, W. P. (2000). Different outcomes of the Wilcoxon-Mann-Whitney test from different statistics packages. *The American Statistician* 54(1): 72-7.

Eklund, A., Nichols, T. E., & Knutsson, H. (2016). Cluster failure: Why fMRI inferences for spatial extent have inflated false-positive rates. *Proceedings of the National Academy of Sciences* 113 (28):7900-5.

Günther, A., & Höfler, M. (2006). Different results on tetrachorical correlations in Mplus and Stata. *International Journal of Methods in Psychiatric Research* 15(3): 157-66.

Thiem, A. (2014). Navigating the complexities of Qualitative Comparative Analysis: Case numbers, necessity relations, and model ambiguities. *Evaluation Review* 38 (6):487-513.
