# Supplementary material for: Algorithmic bias in social research: A meta-analysis
Source: PLoS One. 2020 Jun 8;15(6):e0233625. doi: 10.1371/journal.pone.0233625 (PMC7279593; doi:10.1371/journal.pone.0233625)
Supplement: Attachment — Submitted filename: response_letter.docx [file pone.0233625.s004.docx]

**Response to Reviewers**

We thank the two reviewers for their helpful feedback, and the academic editor of *PLOS ONE* for having given us the opportunity to respond to the reviewers’ comments and to revise the manuscript. In this response letter, we answer to each reviewer comment, explaining how we have addressed the issues raised, or why we did not implement a suggested change. When we did not implement a reviewer’s suggested change, we have made every effort to justify this decision, which we have reached only after careful consideration, in sufficient detail.

**GENERAL CHANGES**

1. For better structuring, we have divided the subsection “Analytical Strategy” into “Analytical Strategy” and “Results”. Previously, the explanation of how we computed algorithmic bias and the results of the meta-analysis were part of the same section.
2. The paragraph on the R function responsible for importing all material and generating all results (algoBias) has now been moved from the subsection “Analytical Strategy” to the subsection “Data”.
3. The number of reanalyzed solution in the meta-analysis has changed slightly (215 now). Appendix A1 did not list six studies yet that were already in the meta analysis, and three studies should not have been in the final set of studies because their respective journals were not listed in the JCR citation reports. This small problem we only noticed after having re-checked all data and replication material very thoroughly again before re-submission. The results did change in consequence by a small absolute percentage difference of 0.1% for two bias categories in Figure 3. The general results are not affected.
4. So as to provide a little more substance to the subsection “Results”, we have now added the example of a study that managed to completely avoid confirmation and data inflation bias, but suffered from large algorithmic bias. As the data for this study are available as part of our replication package, our hope is also that interested readers and possibly also instructors of QCA who would like to incorporate our article in their teaching could use this study for replication and teaching purposes.
5. We have now eliminated the word “publication bias” from the entire text because the concept’s exact definition and content seems to vary too much between researchers.

**REVIEWER 1**

- **Comment 1**: In re-analyzing datasets from published articles, the authors find that a QCA performed with the right algorithm frequently leads to multiple results (for a single dataset). These results are equivalent in the sense that they equally fit the dataset. They argue that it is then impossible for researchers to know which one is a better reflection of the data-generating structure, and that they should thus report all results in noting that they can all be the data-generating structure. In other words, after most QCA, researchers should say “maybe this result is the right one, or maybe it is this other one, I don’t know”. From Figure 3, we see that it isn’t rare that the right QCA algorithm produces more than 10 different results. This finding has important practical implications for QCA users. Assuming that they are looking for the data-generating process, it seems that the risk of not being able to identify it among other potential data-generating processes is very real. This seems to me as yet another case against QCA. In view of this, I have two suggestions:

First, I am encouraging the authors to acknowledge that the possibility of having multiple results fitting equally well the dataset can reduce the appeal of QCA for social scientists.

Second, I am encouraging the authors to discuss the conditions under which QCA with the right algorithm can unequivocally find the data-generating structure (number of cases, number of conditions…). This would be of practical use for QCA users and would somehow balance the case against QCA discussed above.

***Response*:** The fact that multiple explanations often fit a set of data is not unique to QCA, but also exists in regression analysis and experiments (e.g., Eberhardt 2013). However, we believe that the appeal of QCA for social scientists should actually ***increase*** when researchers learn that there is a possibility to always reveal the true model, even if it remains of one several models from which the analyst has to choose. If researchers’ foremost interest is in getting closer to the truth, not in crafting papers that are easy to publish because they tell a strong story (when there may in fact be none in the data), they should always prefer sum irredundancy, and not sum minimality, as the objective function for the QCA optimization algorithm. The problem of many models is sometimes alleviated by essential prime implicants, that is, specific causal paths that appear in every single model. This insight is very valuable and could provide the focus of interpretation in a study whose data fit dozens or more explanations.

With the right solution type and the correct objective function set for the optimization algorithm, QCA will reliably recover the data-generating structure, or at least all parts that are discoverable on the basis of the available data, irrespective of the number of cases or the number of conditions. Of course, non-QCA related problems in data, such as omitted variables, misspecifications, etc. will continue to have a negative impact. These topics, however, would require a separate and dedicated treatment which we think would not fit the scope of our article and be better dealt with separately (in fact, we have a manuscript that addresses the number of exogenous factors ready for submission).

- **Comment 2:** I was wondering what the evolution of the algorithm problem pointed by the authors. It would be interesting to report a figure that shows the proportion of problematic QCA papers by decades. I suspect that the problem is less frequent now than 30 years ago because QCA users increasingly use the QCA R package that uses the right algorithm presented in this paper.

***Response*:** The only software which currently uses the correct objective function by default is the *QCApro* package for *R*, which has only been launched in late 2016. Some of the articles in our data set have used the *QCA* package for *R*, and have been published after the release of *QCApro*. However, they have derived the solution with the incorrect objective function. For example, Kühn and Trinkunas (2017) have not reported 29, 43 other models respectively, for the two outcomes they analyzed. In five to ten years, one could rerun our meta-analysis to check whether Reviewer 1’s intuition that the problem should have disappeared, or at least should have become less serious, proves correct, but currently, it seems to be too early. However, as we make all data and replication material available, every researcher would have the possibility to test this intuition at any time, ideally also with extended data material.

- **Comment 3**: It would be interesting to reflect (perhaps in the conclusion) about the publication bias in QCA. As we all know, not all scientific research gets published. Hence, what the authors observe in their re-analysis of published articles is only a subset of what is happening in QCA research. And the set of published articles is probably different from the set of unpublished studies in many ways. Do they authors have any idea about of how different their result would have been if they had accessed to these unpublished studies? In other words, do they think that the algorithm problem in QCA is more severe or less severe in unpublished studies? My impression is that the problem is less severe in unpublished studies because the studies that report multiple possible results are harder to published than those with only one result (journals and reviewers liking neat results). In other words, there might be more QCA using the right algorithm among the unpublished studies. I’m curious to hear what the authors think about that.

*Response*: This is a very good point, which we have integrated now into Appendix A2, where we describe our data collection strategy in more detail. We have tried several options in including this point in the main manuscript, but finally felt this point was too specific to integrate well there.

It could indeed be that submissions that have used the correct objective function have not been published so far because they had a harder time getting past reviewers and editors. However, due to the recentness of the recognition of this problem, we doubt that the number of unpublished studies that explicitly use the correct objective function in the algorithm is high enough to have a significant impact on our results. On the other hand, there could be a counterbalancing logic, namely when submissions get rejected by methodologically up-to-date reviewers because the authors have used sum minimality as the objective function. Or authors attempt to sell the use of the correct objective function as a strength of their manuscript over previous work (e.g., Andreas *et al*. 2018, 82). In the absence of more concrete information, we cannot say which logic prevails.

**REVIEWER 2**

- **Comment 1**: It would be helpful if the authors could comment on the role of limited diversity in algorithmic bias. Is it likely to exacerbate the problem, or is algorithmic bias unrelated to limited diversity? Are there any other features of the data which may make algorithmic bias more or less likely?

***Response*:** To the best of our knowledge, there is so far no way of directly computing the exact number of models which generalizes to any input function, irrespective of whether it is completely specified or incompletely specified (the later corresponds to what QCA researchers usually call “limited diversity”, and a completely specified function is simply the limiting case of an incompletely specified function). The number of models is a function of the number of prime implicants, which is itself a function of the number of input variables. However, predicting the number of models from either is not straightforward: it ultimately depends on how output values are distributed across different minterms. The range of the number of prime implicants as a function of input variables was first derived in Chandra and Markowsky (1978). Sasao and Butler (2001) have derived the number of irredundant sums, but with two important preconditions: the input function must be symmetric and the number of prime implicants exceeds the number of minterms by one. In other words, these authors’ problem represents a very special case of optimization, and there is a reason why such preconditions are formulated: even for few input variables, formal calculations are computationally very expensive.

If a truth table is complete, the use of sum minimality instead of sum irredundancy as the objective function may still lead to biased results. As an example, take the function

E (A, B, C, D) = ∑f_1_(0,1,2,3,4,6,7,10,12,13,15) + ∑f_0_(5,8,9,11,14),

which has no don’t cares (no unobserved truth table rows). Boolean minimization yields five irredundant sums, two of which are minimal sums:

m_1_: ab + bCd + (ad + ABc + BCD) ↔ E

m_2_: ab + bCd + (aC + ABD + Bcd) ↔ E

m_3_: ab + bCd + (ad + ABD + Bcd + BCD) ↔ E

m_4_: ab + bCd + (aC + ABc + Bcd + BCD) ↔ E

m_5_: ab + bCd + (aC + ad + ABc + ABD) ↔ E

We have improved the manuscript with respect to the topic of limited diversity in the section on inflation bias. In the previous version, we only briefly touched upon this aspect.

- **Comment 2**: The sections “Switching Circuit Optimization with QMC: A Primer” and “Algorithmic Bias” are clearly written and very valuable indeed. The authors’ own empirical analysis is carried out competently given the sample used, but they should make it clear in the main body of the text that the 214 papers used constitute only around 60% of the 360 papers deemed “eligible”. This information is currently only to be found in an online appendix. Accordingly, the authors should moderate their claims regarding the likely extent of the problem of algorithmic bias, given that they cannot be sure whether their 214 papers are representative of all published papers in this area.

***Response*:** This comment is related to Comment 3 by Reviewer 1. Almost all of the papers categorized as eligible but not included in the final data set were excluded because the authors did not respond to our e-mail request for sharing the data (no e-mail was returned because the address was not valid anymore), or because the information necessary to rerun the analysis could not be restored even after contact with the authors. In a few cases, authors also explicitly refused to share their data. In the absence of reliable information, we thus cannot clearly say whether the numbers we report on the basis of the 215 (see General Changes, 3.) included articles are possibly too low. However, we have noticed this potential problem now also in Appendix 2, where we explain our data collection strategy in detail. Again, we hope that since we make all data material available to the scientific community, future studies might have the possibility to test for these potential problems.

- **Comment 3**: The authors should explain how they arrived at their method of computing publication bias (pp.15/23). If they did so, it might become clearer why a compound measure of three separate types of bias is needed or helpful – this is currently not clear. All three types of bias included in the measure are complex and it may not be possible (or sensible) to capture them within a single indicator. Confirmation bias is a real phenomenon, but the authors do not allow for the possibility that a researcher’s preference for a particular model may actually be justified because it is based on sound theoretical analysis. Inflation bias can only arise if there is limited diversity in the data, so it is not relevant to all studies. Both confirmation bias and inflation bias are important topics in their own right, but without detailed discussion and analysis, their inclusion in the overall measure of publication bias seems slightly arbitrary, not least because there are likely to be other sources of publication bias in addition to the three included here. If the authors do wish to measure algorithmic bias, a more transparent way of doing so would be to develop a single, one-dimensional measure for it.

***Response*:** In order to isolate algorithmic bias, we needed to factor out all other sources of problems that could lead to a situation where fewer models are reported than do fit the data. Generally, this is easy to measure since we know the number of models reported and the number of models that do fit the data equally well. Of ultimate interest to us, however, is not the overall measure of publication bias (see General Changes, 5.) as derived by the difference between the total number of models and the number of models reported, but only that part of this difference which is attributable to the use of the incorrect objective function. We have tried to reformulate the concerned passage on p.15 in the hope that it will have become clearer now.

- **Comment 4**: Linked to the last point I made under “technical issues” is a wider issue concerning conceptual clarity, which is that algorithmic bias is actually not the same thing as publication bias. Publication bias arises from a tendency of submitting to and acceptance by journals of positive results only. The authors themselves acknowledge that these are different points in their introduction where they note that publication bias arises from selective reporting which is committed consciously, unlike algorithmic bias of which authors are not even aware. So while publication bias is an important topic, the term should not be widened unnecessarily.

***Response*:** See General Changes, point 5.

- **Comment 5**: The authors claim that algorithmic bias contributes to the “reproducibility crisis” without any supporting evidence. They certainly do not provide any evidence as to why algorithmic bias “may be one of the largest contributors to irreproducible research and publication bias in some areas of science”, p.2/23, thus this appears to be mere speculation.

***Response*:** We have now added Eklund *et al*. (2016) a reference that provides such evidence. It is one of the few other existing studies that link algorithms implemented in software to the validity and reproducibility of research findings. And we have reformulated the concerned passage in that the quoted statement has become less strong than it was before.

- **Comment 6**: Likewise, the authors claim that problems arise when methods travel across disciplinary boundaries. Indeed, this appears to be the case with algorithmic bias which only arises in a social science context but which is irrelevant in the context of electric engineering where the Quine-McCluskey algorithm was first developed. But the authors provide no analysis of why such problems should arise more generally whenever methods travel across disciplinary boundaries. Granted, a careful analysis of this possibility should be undertaken when methods are employed in a novel context, but the danger with the authors’ general warning is that researchers may become adverse to employing methods from other areas altogether. But this would potentially deprive them of valuable innovative tools.

***Response*:** In the best case, at least from our perspective, more thorough testing would be carried out in the future before relying on tools that have been developed in very different contexts and for very different purposes. That should also be in the importers’ own interest. For example, in risk and safety assessment it is common practice to import methods initially developed for very different purposes. For example, human reliability assessment tools developed for the nuclear industry are adapted to quantifying human performance in the context of air traffic management, process safety, railway safety management, radiotherapy patient safety (see e.g., Pandya et al. (2018), Kirwan B & Gibson (2007)). However, every single detail of the method (e.g., risk/safety indicators, base probability values, risk attribute levels, task types) is adjusted to the new domain.

As the public finances science, it will also have much more trust in scientific findings if it knows that scientists have done their best in making sure the findings they produce stand on a foundation as solid as currently possible, or if revelations about shaky research do not appear in the news as often as over the last few years (e.g., Stapelgate, Climategate, etc.). That will increase the “costs” of introducing new techniques, but the long-term costs of unsolid research resulting from the premature use of an unsuitable technique may be considerably higher. The implications of this reasoning should be evaluated positively: if a researcher wants to use techniques from electrical engineering, for example, that researcher should make sure that electrical engineers get involved as well so as to reduce the risk of employing a tool that is not well-understood or ill-applied in another context. In other words, interdisciplinarity will be encouraged, ideally to the benefit of all involved parties.

- **Comment 6**: Similarly, the paper’s title itself is misleading: algorithmic bias is a serious problem well worth the authors’ thorough discussion, but it is not a problem for all of social science.

***Response*:** We do not imply that all social research has problems with algorithmic bias. In reference to the title “Algorithmic Bias in Social Research”, we merely analyze ***whether*** there exists algorithmic bias somewhere in social research. The title leaves it open whether algorithmic bias exists elsewhere in the social sciences than those three bodies of QCA literature from management, political science and sociology we study, but it does not imply that this is the case. We would surmise that algorithmic bias in QCA is not a unique phenomenon, and other areas ***may*** have experienced similar problems without being aware of this. In this way, our article provides an analysis of algorithmic bias in a specific body of literature, but it would like to alert researchers to the ***possibility*** that algorithmic could also be a problem other areas. For instance, some machine-learning algorithms may be ill-employed, or some piece of software may have routines that are suitable for some problems, but not for others, without users being aware of that.

**REFERENCES**

Andreas, Jan-Justus, Charlotte Burns, and Julia Touza. 2017. “Renewable Energy as a Luxury? A Qualitative Comparative Analysis of the Role of the Economy in the EU's Renewable Energy Transitions During the ‘Double Crisis’.” *Ecological Economics* 142:81-90.

Chandra, A.K. and Markowsky G. 1978. “On the number of prime implicants.” *Discrete Mathematics* 24:7–11.

Eberhardt, Frederick. 2013. “Experimental indistinguishability of causal structures.” *Philosophy of Science* 80 (5):684-96.

Eklund, Anders, Thomas E. Nichols, and Hans Knutsson. 2016. “Cluster failure: Why fMRI inferences for spatial extent have inflated false-positive rates.” *Proceedings of the National Academy of Sciences* 113 (28):7900-5.

Goodman, Steven N., Daniele Fanelli, and John P. A. Ioannidis. 2016. “What does research reproducibility mean?” *Science Translational Medicine* 8 (341):341.

Kühn, David, and Harold Trinkunas. 2017. “Conditions of military contestation in populist Latin America.” *Democratization* 24 (5):859-80.

Sasao, Tsutomu, and Jon T. Butler. 2001. “Worst and best irredundant sum-of-products expressions.” *IEEE Transactions on Computers* 50 (9): 935-948.

Pandya, D., Podofillini, L., Emert, F., Lomax, A. J., & Dang, V. N. 2018. Developing the foundations of a cognition-based human reliability analysis model via mapping task types and performance-influencing factors: Application to radiotherapy. *Journal of Risk and Reliability*, *232*(1), 3-37.

Kirwan B and Gibson WH. CARA: A human reliability assessment tool for air traffic safety management— technical basis and preliminary architecture. 2007. In: Redmill F and Anderson T (eds) The safety of systems. London: Springer, pp.197–214.
